# Multiscale community detection in Cytoscape

**DOI:** 10.1371/journal.pcbi.1008239

**Published:** 2020-10-23

**Authors:** Akshat Singhal, Song Cao, Christopher Churas, Dexter Pratt, Santo Fortunato, Fan Zheng, Trey Ideker

**Affiliations:** 1 Department of Computer Science and Engineering, University of California, San Diego, La Jolla, California, United States of America; 2 Department of Medicine, University of California, San Diego, La Jolla, California, United States of America; 3 School of Informatics, Computing, and Engineering, Indiana University, Bloomington, Indiana, United States of America; National Center for Biotechnology Information (NCBI), UNITED STATES

## Abstract

Detection of community structure has become a fundamental step in the analysis of biological networks with application to protein function annotation, disease gene prediction, and drug discovery. This recent impact creates a need to make these techniques and their accompanying visualization schemes available to a broad range of biologists. Here we present a service-oriented, end-to-end software framework, CDAPS (Community Detection APplication and Service), that integrates the identification, annotation, visualization, and interrogation of multiscale network communities, accessible within the popular Cytoscape network analysis platform. With novel design principles, CDAPS addresses unmet new challenges, such as identifying hierarchical community structures, comparison of outputs generated from diverse network resources, and easy deployment of new algorithms, to facilitate community-sourced science. We demonstrate that the CDAPS framework can be applied to high-throughput protein-protein interaction networks to gain novel insights, such as the identification of putative new members of known protein complexes.

This is a *PLOS Computational Biology* Software paper.

## Introduction

One of the fundamental features of a complex network is the notion of community, which can be defined as a group of nodes that are more densely connected with each other than they are to the rest of the network. Community detection is a class of pattern recognition methods that assign network nodes to groups, or communities, based on the network‘s structural organization. As an important technique to probe the structural organization of a complex network, it has been successfully applied to many problem domains in systems biology, such as identifying protein complexes[1–4], cataloging ‘omics profiles[5–8], and prioritizing new disease genes[9–11].

Clustering, which relates to community detection at a single scale, is a well-established technique in network analysis and is supported by many applications such as Clustermaker2[12], CytoCluster[13], ClusterViz[14], and MCODE[15], which have been made available in bioinformatics environments like Cytoscape[16]. Recent work in multiscale network community detection makes it possible to build hierarchical representations of biological structure and function directly from networks[17–19]. These developments create a new challenge: to make multiscale community detection techniques and their accompanying visualization schemes available to a broad range of biologists.

**Fig 1:**
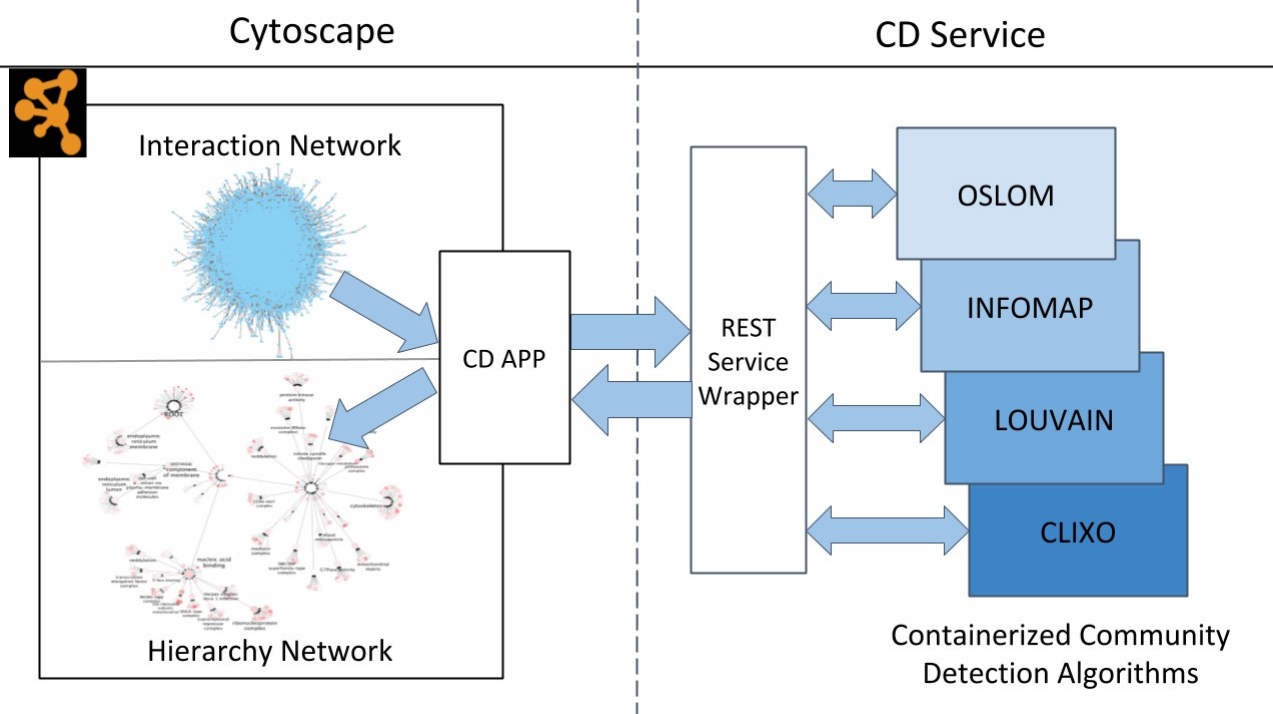
Overview of the CDAPS workflow. A flowchart illustrating various components of the CDAPS framework and the data flow between them. The CD app can send edges of an interaction network for community detection to the CD service. The service then launches the corresponding Docker image which executes the required task and sends back a hierarchy network to the CD app for visualization and community labeling.

Here, we present a software infrastructure to address these challenges, termed CDAPS (Community Detection APplication and Service), deployed as an App in the Cytoscape platform for network analysis. CDAPS has several novel aspects in its design relative to existing applications in the field of bioinformatics. First, beyond assigning nodes in an input network to a set of clusters, multiscale community detection in CDAPS summarizes a network’s underlying multiscale structure in a separate hierarchical ontology, where each node represents a community. Second, CDAPS invokes community detection and functional enrichment algorithms via REST (REpresentational State Transfer) queries to a remote service outside of the Java-based Cytoscape desktop environment (**Fig 1**). On that service, community detection algorithms are encapsulated using Docker containers[20], enabling them to run in their native language and environment. Further, the use of remote servers allows incorporation of new algorithms without the need to update the App, and it removes the computational overhead of running these algorithms in the Cytoscape desktop application. As a whole, CDAPS provides an end-to-end pipeline that creates, annotates, visualizes, and interrogates hierarchical models based on multiscale patterns in networks.

## Design and implementation

The CDAPS framework contains two major components: CD App and CD service. CD App is part of the Cytoscape desktop application and acts as a client to the CD service. The CD service, as a typical service-oriented architecture [21], performs the community detection computation on a remote server. In the CD App, the user chooses a community detection algorithm to be applied to a given *interaction network* and then the pertinent data is sent to the CD service, where a Docker image hosting the algorithm is invoked to produce a hierarchical network. The results are returned to the CD App, which generates a new *hierarchy network*, or ontology, which contains information of the resulting communities (as nodes) and their hierarchical relationships (as edges). The whole framework provides efficient interfaces among different modular components of the pipeline (**Fig 1**).

### CD App

CD App is an App for Cytoscape [16] which acts as a client for the CD service, providing a convenient user interface through menu options. Below we briefly describe its functions:

1. **Community detection**: In this initial release, we provide four algorithms: Louvain [22], Infomap [23,24], OSLOM [25], and CliXO [26]. Louvain, Infomap, and OSLOM are popular community detection algorithms which are highly scalable and capable of organizing multiscale communities as a hierarchy. We also include our previously described CliXO algorithm, which uniquely organizes communities hierarchically in the form of a directed acyclic graph (DAG), i.e. a community is allowed to be part of different overlapping communities. These algorithms have previously been implemented without graphical user interfaces, making it difficult for new users to tune their parameters. Through CDAPS, we provide an interactive interface to tune parameters, such as the “resolution parameters” in Louvain (via Reichardt-Bornholdt configuration model [27] implemented in http://github.com/vtraag/louvain-igraph), Infomap (via the Markov time parameter in the Infomap package [28]), and OSLOM that control the granularity of the resulting communities. Because community detection is an active research field, and many new algorithms are proposed every year [29], the CD app is designed to be easily extended by packaging new algorithms as Docker images in a standardized format and adding them to the CD service (see details in "CD service").
2. **Functional enrichment**: One of the key downstream analyses of community detection is to annotate the resultant protein communities by determining their overlap with gene sets associated with known functional or biological entities. We currently provide interfaces with the following external functional enrichment tools: g:Profiler [30], Enrichr [31], and iQuery (iquery.ndexbio.org). These tools can be applied to all or a subset of communities.
3. **Interaction sub-networks**: We also provide a feature to enable interrogation of the communities in the hierarchical model by retrieving the subgraph of the interaction network corresponding to the genes in a given community.

Functional enrichment and sub-network interrogation can be invoked by menu options associated with any node (i.e. a community) in the hierarchy network. The CDAPS framework considers a hierarchy network and its source interaction network as two separate entities. We link these networks by keeping a simple reference to the source interaction network as an attribute of the hierarchy network. For a given interaction network, users can generate many alternative hierarchies with different algorithms and parameter settings, and they can save and manage them independently. For reproducibility, the CD App stores the name of the algorithms and the accompanying parameters as an attribute of the hierarchy network.

### CD service

CD service is a REST web service that acts as a bridge between the Cytoscape desktop app and the hierarchical community detection algorithms. The service provides a platform-independent endpoint that is accessible to CD App and other tools such as a website or Jupyter notebook [32]. To facilitate easy installation and reproducibility, community detection algorithms are packaged as Docker images that are called by the CD Service on remote servers (**Fig 1**). An additional advantage of using remote servers is to avoid problems on platforms that do not support direct invocation of Docker images.

The infrastructure of the CD service facilitates updates and extends the capabilities of the CD app by enabling new Docker images to be incorporated into the CD service to support additional algorithms. Updates to the CD service are transparent to users because the CD App automatically updates its interface by querying the service to find the latest algorithms and their parameters. In addition to the community detection algorithms, the CD Service interfaces to external functional enrichment tools to find annotations that can help label a community and provide insight into its function (see the “CD App” section above).

## Results

As proof of concept, we explored a workflow for creating a hierarchical model from a large protein interaction network in Cytoscape. Without loss of generality, we chose to use OSLOM [25] as the community detection algorithm and BioPlex 2.0 [4] as the interaction network. BioPlex is a physical protein-protein interaction network containing 10,961 proteins and 56,553 interactions determined by the affinity-purification mass-spectrometry (AP-MS) technique. The goal of the analysis was to reveal meaningful structures of this complex network, as well as to discover *de novo* protein modules or novel extensions of previously identified protein modules. We then used a functional enrichment tool, g:Profiler [30], to generate labels for communities based on significant overlap with gene sets in databases. We describe the steps of this workflow as follows.

**Fig 2:**
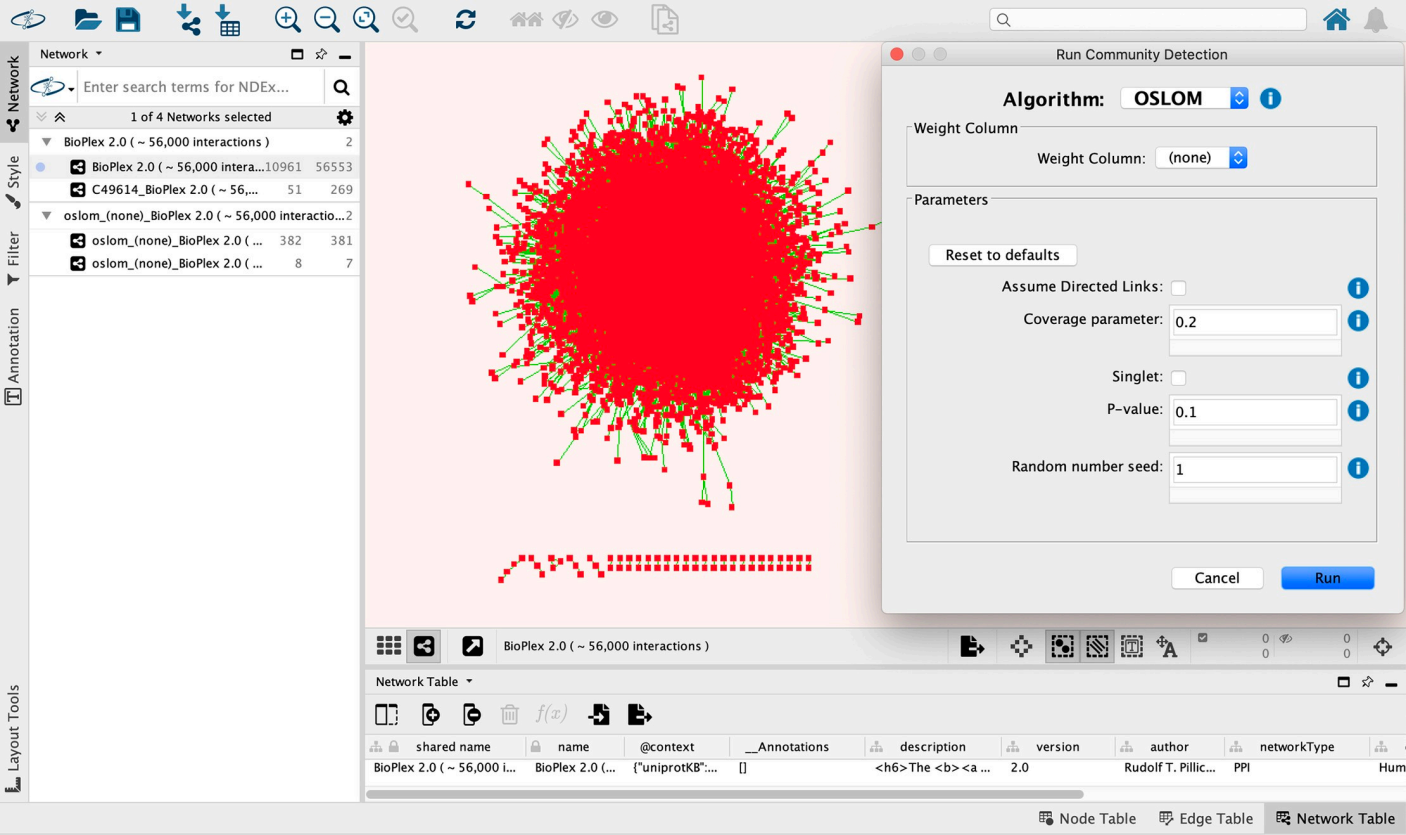
Run community detection on a protein-protein interaction network. A Cytoscape example screenshot showing the BioPlex 2.0 network [4] imported from NDEx (http://doi.org/10.18119/N91597), along with settings for running OSLOM [25]. Related to steps 4–5 in the example workflow.

**Fig 3:**
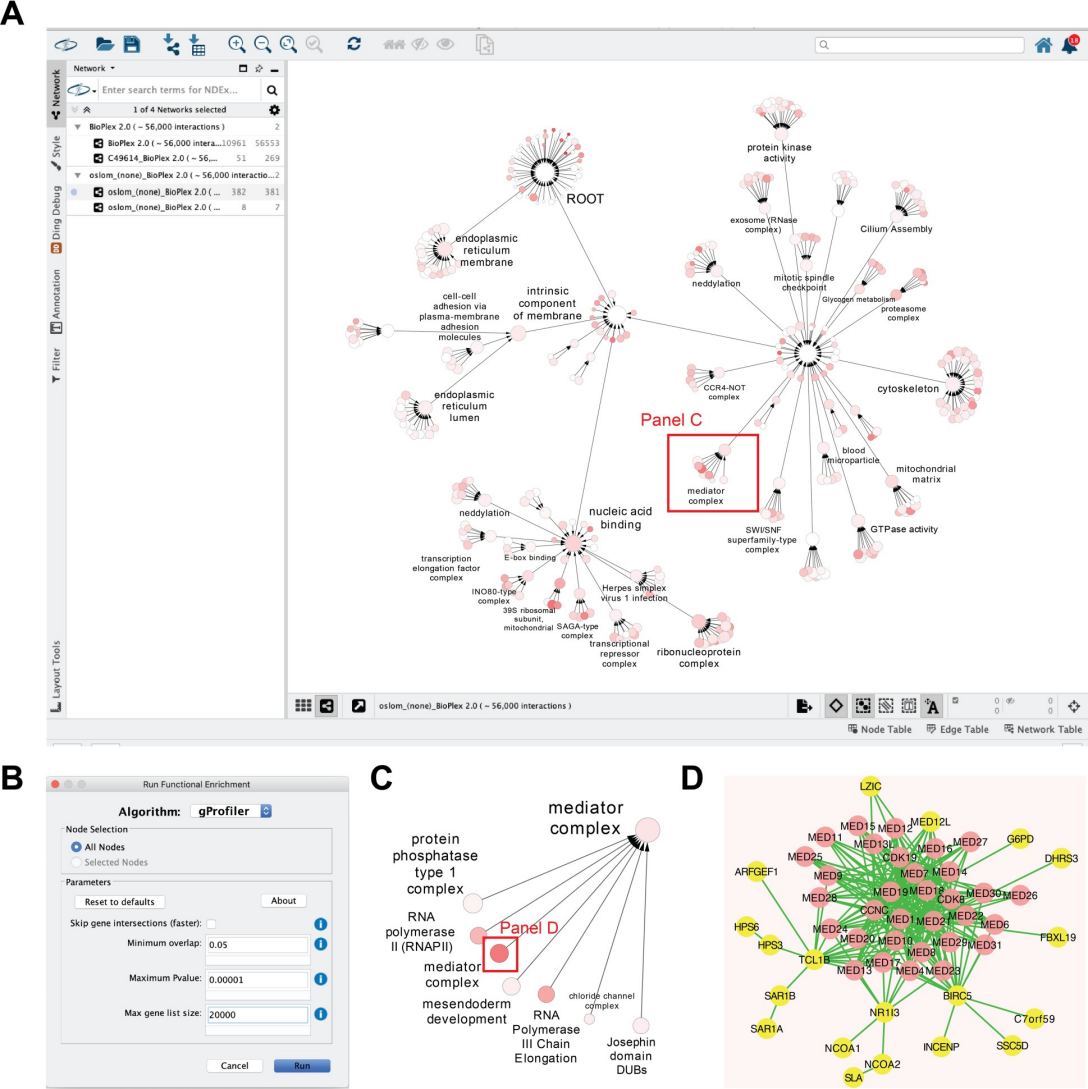
Results of hierarchical community detection and downstream analyses. (A) A hierarchy derived from the BioPlex 2.0 [4] interaction network with OSLOM [25] and annotated by g:Profiler. *Red rectangle*: a subregion in the hierarchy related to the mediator complex. (B) The menu to set up functional annotation with g:Profiler. (C) A zoom-in view of the sub-hierarchy highlighted in panel A. *Red rectangle*: a community to be further explored. (D) The interaction subnetwork of the community highlighted in panel C. Proteins, not known as members of the mediator complex, are highlighted (*yellow*). Related to steps 6–11 in the example workflow. *Node color*: The overlap (Jaccard Index) between a community and the gene set corresponding to its annotation (panels A and C). Networks can be found on NDEx: http://www.ndexbio.org/#/network/380d5904-bd9f-11ea-aaef-0ac135e8bacf (panel A). http://www.ndexbio.org/#/network/28fdd535-bbe5-11ea-aaef-0ac135e8bacf (panel D).

1. Download and install Cytoscape from http://www.cytoscape.org/.
2. Start Cytoscape and instantiate a new session.
3. Install the app. **Apps > App Manager > Select “CyCommunityDetection” > Install**.
4. Import the example network. Click **NDEx icon > Import Network from NDEx > Search “BioPlex” > Select "BioPlex 2.0 (56,000 interactions)" > Close.** The DOI of this network is http://doi.org/10.18119/N91597.
5. Execute OSLOM for BioPlex (**Fig 2**). Click **Apps > Community Detection > Run Community Detection > Select “OSLOM” from the algorithm drop-down > Set “coverage parameter” to 0.2 > Set “random number seed” to 1 > Run**. This step creates a hierarchy network. Note that community detection algorithms are in general stochastic, and thus they may generate different results if the random seed parameter is not fixed. The parameters of community detection, including (if applicable) the random seed, are stored as a property of this hierarchical network to ensure the reproducibility of this workflow (column “description” of network table).
6. Run functional enrichment on the hierarchy network. Click **Apps > Community Detection > Run Functional Enrichment > Select g:Profiler from the algorithm drop-down > Set “minimum overlap” to 0.05 > Run.** This step adds annotations to the hierarchy network (**Fig 3A and 3B**).
7. Without loss of generality, with the hierarchy network in the viewport, type “mediator” in the search box at the upper-right corner of Cytoscape to highlight the two nodes annotated as “mediator complex”. This is because the “mediator complex” is the best-matched gene set in reference databases of g:Profiler for both communities (**Fig 3C**). Between these two communities, the smaller one with 51 genes better matches the reference version of the “mediator complex” (CORUM:230; Jaccard index 0.64; relevant information can be found in columns “CD_AnnotatedMembers_SourceTerm” and “CD_AnnotatedMembers_Overlap”).
8. For this node, copy the content in the attribute “CD_NonAnnotatedMembers” in the “Node Table” of the hierarchy network, which represents a subset of proteins in the community that is not recorded in the database entry of “mediator complex” queried by g:Profiler (i.e. CORUM:230 in this example).
9. **Right-click the node > Apps > Community Detection > View Interactions for Selected Node.** It creates a sub-network of the BioPlex 2.0, which contains only the proteins in this community (51 nodes, 269 edges) (**Fig 3D**).
10. With the interaction sub-network in the viewport, paste the string copied from Step 8 in the search box above the upper right corner of the viewport. It highlights some nodes in the interaction sub-network.
11. From the results, it can be noticed that several proteins that have not been known as members of the mediator complex (e.g. BIRC5, LZIC, NR1I3, TCL1B) have many interactions with known mediator complex members [33] (**Fig 3D**). These proteins are putative novel components of the mediator complex based on the BioPlex 2.0 network.

In support of their association with Mediator, BIRC5 and NR1I3 have reported roles as transcriptional activators [34,35], in agreement with the function of the Mediator complex which transduces signals from transcriptional activators bound to enhancers to the basal transcription machinery (including RNA polymerase II) [33], suggesting that these physical interactions are functionally relevant.

To test the performance of the CDAPS framework, we ran three community detection algorithms, Louvain, Infomap, and OSLOM [22,23,25], available through CDAPS, on popular biological networks and recorded the time taken by the algorithms inside the CD service along with the runtime corresponding to overhead (time taken transmitting the network and visualizing/constructing the resultant hierarchy). We observed that the fraction of time spent on overhead decreases sharply as the total runtime increases, suggesting that our implementation of CDAPS efficiently integrates the algorithms into the Cytoscape interface. For instance, for the Bioplex 2.0 network [4], the total runtime with OSLOM is about 43.85 seconds, including the CDAPS overhead time of 0.99 seconds, 2.3% of the total runtime (**Table 1**). In general, for any network for which the total runtime exceeds 50 seconds, the time overhead of running a community detection algorithm through CDAPS has a median of less than 1%. The CD service is currently hosted on an Intel(R) Xeon(R) E5-2687W v3 processor with 32 GB of memory. All the Docker containers are run with the default configuration and, thus, each container has all the resources of the host processor.

**Table 1:** Run-time performance of CDAPS.

Input Network (*#nodes/ #edges*)	Louvain Runtime (T1*/T2^†^/T3^‡^ in s)	Infomap Runtime (T1*/T2^†^/T3^‡^ in s)	OSLOM Runtime (T1*/T2^†^/T3^‡^ in s)
Drugbank Database—v4.1 (*7*,*093/13*,*239*)	1.09/2.16/2.31	0.97/1.10/1.48	20.79/21.21/21.60
BioPlex 2.0 (*10*,*961/56*,*553*)	1.58/2.09/2.38	1.35/2.17/2.70	42.86/43.30/43.85
BioGRID: Protein-Protein Interactions (D. melanogaster) (*9*,*337/61*,*188*)	1.92/2.19/2.39	3.26/4.18/4.42	73.15/73.40/73.65
HumanNet—PI (*15*,*351/158*,*499*)	2.60/3.18/3.38	3.34/4.21/4.62	382.15/382.66/383.23
STRING—Human Protein Links—High Confidence (Score > = 0.7) (*17*,*185/420*,*534*)	4.01/4.39/4.63	11.57/12.42/12.89	191.02/192.25/193.97
Human Gene Regulatory Network of Mesothelioma (*17*,*490/631*,*556*)	8.03/8.56/8.88	20.35/21.58/22.11	421.13/422.28/422.80
DisGeNet—Gene-Disease-PMID Associations (*41*,*710/1*,*573*,*979*)	11.76/13.29/13.88	14.80/16.15/17.10	922.33/924.54/940.62

*T1 indicates the time taken by a community detection algorithm inside the CD Service.

^†^T2 indicates T1 plus the network transmission time to and from the CD Service.

^‡^T3 indicates T2 plus network creation time in Cytoscape.

## Availability and future directions

Flexibility and extensibility are the two main strengths of the CDAPS framework, allowing us and others to continue to grow and evolve the repertoire of community detection algorithms available to users. The use of Docker images to encapsulate algorithms will reduce the need for customization and simplify their deployment to the service. As new algorithms are deployed to CDAPS, the CD App will fetch the list of currently available algorithms from the CD service and update the CD app interface in Cytoscape without requiring any action on the part of users.

To enable a web-based, interactive investigation of hierarchical models of network communities, a compelling future direction is to create a pipeline that connects the CDAPS to our HiView platform [36]. HiView provides an intuitive visualization of hierarchical/nested structures with a powerful “circle packing” layout [37], as well as the ability to use any community-defined gene list to probe the support of the same community in orthogonal network datasets hosted on the NDEx database [38].

We will also apply workflows that use the results of community detection to select particular communities of high interest, such as those that significantly aggregate the disease-related statistical signals of individual genes/proteins extracted from large-scale genomic studies including GWAS (Genome-Wide Association Studies) [11,39] and cancer whole-exome sequencing.

The source code of CD App, along with the user documentation, is available at https://github.com/cytoscape/cy-community-detection. CD App can be directly installed through the Cytoscape Application Manager. The codebase of CD service and its documentation is available at https://github.com/cytoscape/communitydetection-rest-server.

## Supporting information

S1 File Bioplex CDAPS cytoscape session. This file contains the Bioplex 2.0 interaction network, the derived hierarchy network, and the interaction sub-network used in the worked example.
(CYS)

S2 File CD App binary. The java archive file can be directly installed in Cytoscape.
(JAR)

S3 File CD App user document. This file contains the steps required to compile CD App source code, install the binary in Cytoscape, and use the application.
(PDF)

S4 File Test data. This file contains a list of popular networks, publicly available through NDEx, that were used to test the CDAPS framework. Each test in the file provides the details of parameters used for that particular test and the NDEx URLs for both the input and output networks.
(PDF)
