# Supplementary material for: Multiscale community detection in Cytoscape
Source: PLoS Comput Biol. 2020 Oct 23;16(10):e1008239. doi: 10.1371/journal.pcbi.1008239 (PMC7584444; doi:10.1371/journal.pcbi.1008239)
Supplement: S3 File — This file contains the steps required to compile CD App source code, install the binary in Cytoscape, and use the application. (PDF) [file pcbi.1008239.s003.pdf]

Community Detection App for Cytoscape

Cytoscape is an open source software platform for visualizing complex networks and integrating these with any type of attribute data. The Community Detection App is a Java plugin for Cytoscape which provides the user interface to execute several community detection algorithms and gene-set enrichment tools. All these algorithms and tools run on a remote server which exposes REST API. The app uses these API to execute the algorithms and tools.

1 Source Code Compilation

The source code is available at https://github.com/cytoscape/cy-community-detection.

1.1 Requirements to build

- Java 8+ with jdk
- Maven 3.4 or above

1.2 Building manually

Commands below assume Git command line tools have been installed.

1. git clone https://github.com/idekerlab/cy-community-detection
2. cd cy-community-detection
3. mvn clean test install

The above commands create a binary under target folder as cy-community-detection-<VERSION>.jar, that can be installed in Cytoscape.

2 Binary Installation

2.1 Cytoscape

- If you don't have Cytoscape installed on your system, please follow the instructions at https://cytoscape.org/download.html.
- More information on Cytoscape can be found at https://cytoscape.org.

2.2 Community Detection App

1. Click on Apps → App Manager → Search "CyCommunityDetection" → Install.

3 User Options

The community detection app adds menu options in the menu bar and the context menu. A menu bar is the list of options on the top-left panel of Cytoscape window (Figure A(I)). A context menu gets populated when a user right-clicks on the network view panel (Figure A(II)). In general, the menu bar options of this app apply to the whole network and those in the context menu apply to the selected node(s). The app currently provides three major functions:

3.1 Community Detection algorithms

There are several algorithms which can be used for community detection of a network. However, most of these algorithms are written in languages other than Java and hence cannot be used via Cytoscape. The CD service exposes REST API to execute community detection algorithms and the application acts as a simplified interface between a user and CD service.

A sample workflow to execute an algorithm can be executed as follows: Apps → Community Detection → Run Community Detection → Select an algorithm from the algorithm drop-down → Set parameters of your choice → Run (Figure B).
A short description of each algorithm can be found by clicking on the ”About” button in the launcher dialog. Clicking the ”i” icon displays a brief description of the parameter beside it (Figure B).

3.2 Gene-set Enrichment

One of the main reasons to apply a community detection algorithm on a protein-protein interaction network is to find out previously unknown interactions in known complexes. In order to find known complexes associated with the communities in a hierarchy network, the app currently supports three enrichment tools: g:Profiler, Enrichr, and iQuery. There are two ways of running these tools:

- Menu bar - The menu bar option runs for the whole hierarchy network.
  Steps: Apps → Community Detection → Run Functional Enrichment → Select an enrichment from the algorithm drop-down → Set the desired parameters → Run (Figure A(I), C(II)).
- Context Menu - Another way to call an enrichment tool is through the context menu. However, by default, this option works for selected nodes.
  Steps: Right-click the node → Apps → Community Detection → Run Functional Enrichment (Figure A(II)).

3.3 Interaction Subnetwork

Another useful feature of this application is to view interactions among the genes of a community. This is achieved by creating a sub-network of the original interaction network. The subnetwork contains genes of the selected community node and the edges connecting those genes.
Steps: Right-click the node → Apps → Community Detection → View Interactions for Selected Node (Figure A(II), C(III), C(IV)).

An important point to note about the subnetwork creation task is that it depends on network table (_CD_OriginalNetwork) and node table (CD_MemberList) entries. To create a correct subnetwork both these entries should exist and entry under _CD_OriginalNetwork should point to the original interaction network.

I

II

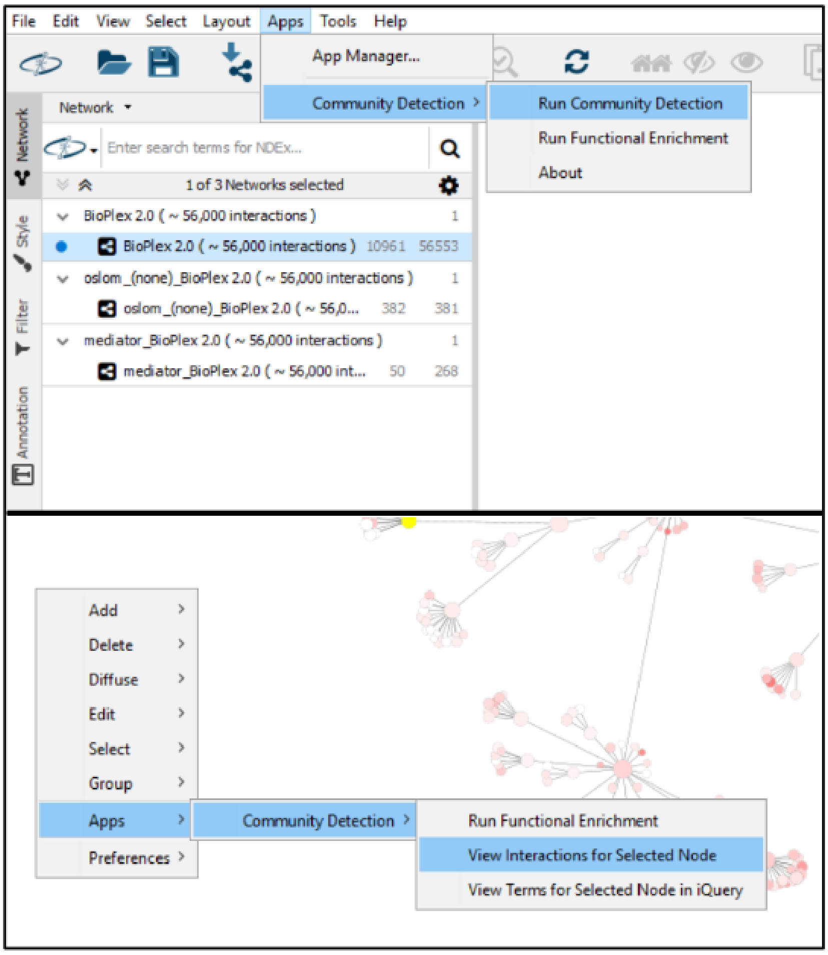

Figure A: Menu options available with the CD App.

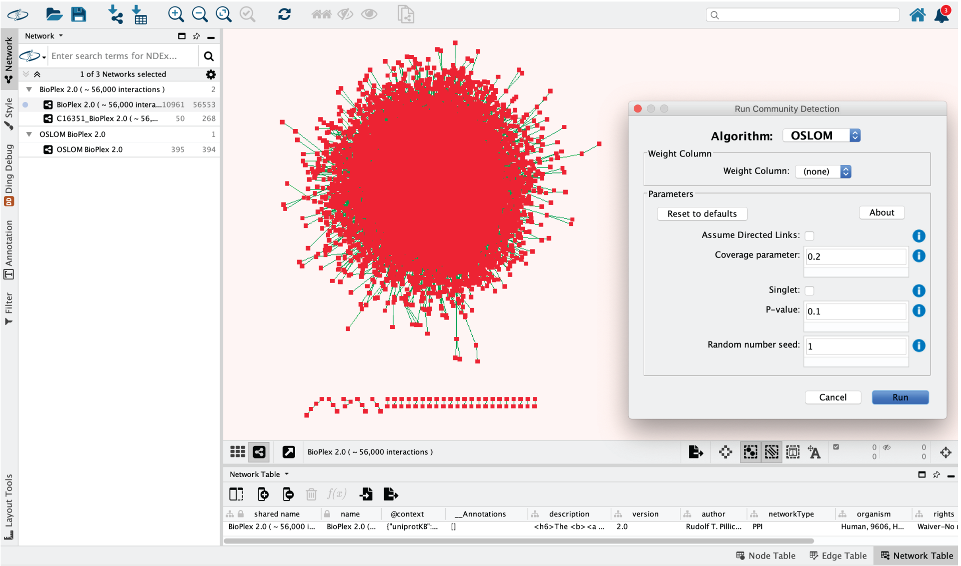

Figure B: A Cytoscape example screenshot showing an interaction network, along with settings for running a community detection algorithm.

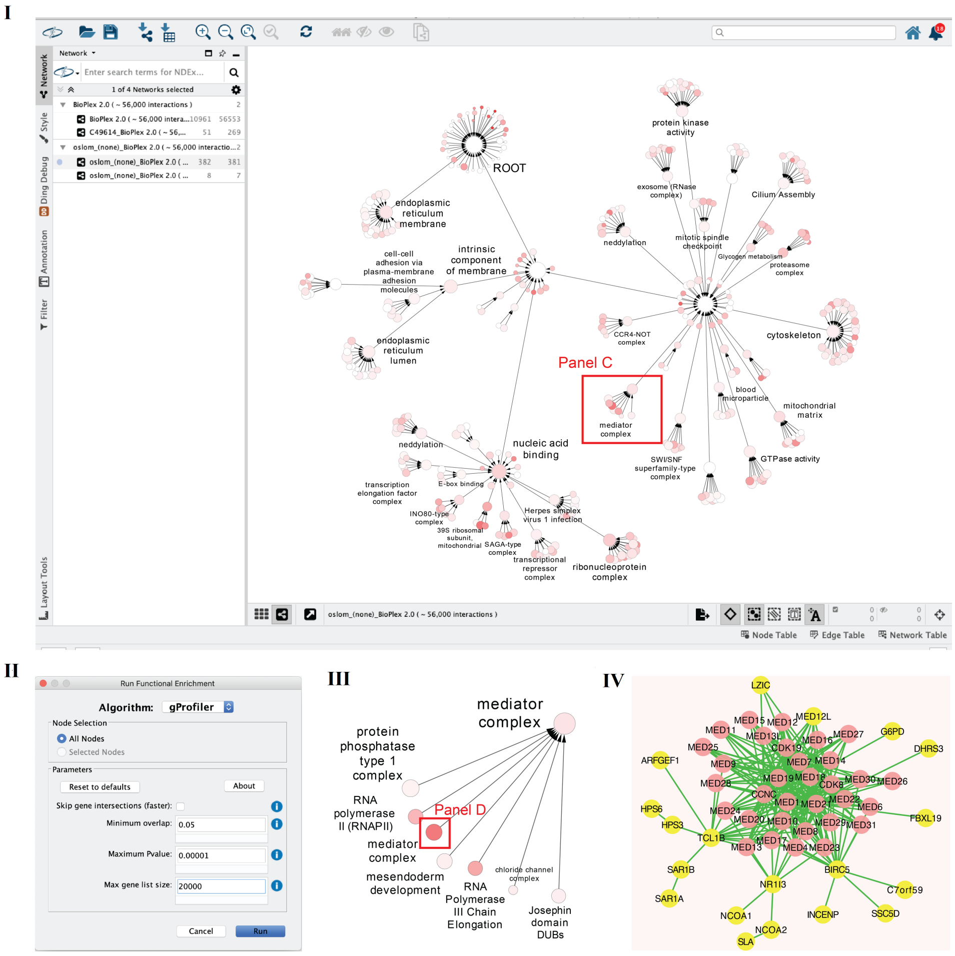

Figure C: (I) A derived hierarchy. (II) Menu to setup an enrichment tool. (III) Zoom-in view of a sub-hierarchy. (IV) The interaction subnetwork of the community highlighted in panel III.
