# Supplementary material for: Multiscale community detection in Cytoscape
Source: PLoS Comput Biol. 2020 Oct 23;16(10):e1008239. doi: 10.1371/journal.pcbi.1008239 (PMC7584444; doi:10.1371/journal.pcbi.1008239)
Supplement: S4 File — This file contains a list of popular networks, publicly available through NDEx, that were used to test the CDAPS framework. Each test in the file provides the details of parameters used for that particular test and the NDEx URLs for both the input and output networks. (PDF) [file pcbi.1008239.s004.pdf]

Test data

Both the networks we chose as test inputs to CDAPS and the resultant hierarchies with parameter settings (relative to default) described below are publicly available on NDEx. Networks were selected according to their popularity and compatibility with community detection algorithms.

1. DrugBank Database - v4.1
    a. **Louvain** [generate true hierarchy: true, random number generator seed: 1, cluster resolution: 10]
    g:Profiler [default settings]
    http://www.ndexbio.org/#/network/53c22b66-7c60-11ea-aaef-0ac135e8bacf
    b. **Louvain** [generate true hierarchy: true, random number generator seed: not set, cluster resolution: 5]
    iQuery [default settings]
    https://ndexbio.org/#/network/3f3b7d1c-7c81-11ea-aaef-0ac135e8bacf
2. BioPlex 2.0
    a. **OSLOM** [coverage parameter 0.2, random number seed: 1, P-value: 0.1]
    g:Profiler [default settings]
    http://www.ndexbio.org/#/network/89cbed2d-599a-11ea-bfdc-0ac135e8bacf
    b. **OSLOM** [coverage parameter 0.5, random number seed: -1, P-value: 0.05]
    iQuery [default settings]
    https://ndexbio.org/#/network/3118eee1-7c8a-11ea-aaef-0ac135e8bacf
3. ProteomeHD
    a. **Infomap** [weight column: none, markov time: 0.75, random number generator seed: 3]
    g:Profiler [skip gene intersections: true, other default]
    http://www.ndexbio.org/#/network/cee1ed12-7e1f-11ea-aaef-0ac135e8bacf
    b. **Infomap** [weight column: score, markov time: 0.9, random number generator seed: None]
    iQuery [default settings]
    http://www.ndexbio.org/#/network/1fce8527-7e21-11ea-aaef-0ac135e8bacf
4. YeastNet_Top300Nodes_wtNames
    a. **CliXO** [weight column: Combined_Score, beta: 0.5, alpha: 0.1]
    g:Profiler [default settings]
    http://www.ndexbio.org/#/network/5ddf6e47-7e18-11ea-aaef-0ac135e8bacf
    b. **CliXO** [weight column: Combined_Score, beta: 0.2, alpha: 0.05]
    iQuery [default settings]
    http://www.ndexbio.org/#/network/cb9d581e-7e19-11ea-aaef-0ac135e8bacf
